# *N*,*N*,*N*′,*N*′-Tetra­methyl­ethylene­diammonium tetra­chlorido­zincate

**DOI:** 10.1107/S1600536813029802

**Published:** 2013-11-06

**Authors:** Muhammad Akhtar, Mohammed Fettouhi, Maqsood Ahmed, Islam Ullah Khan, Saeed Ahmad

**Affiliations:** a Department of Chemistry, Quaid-i-Azam University, Islamabad, Pakistan; b Department of Chemistry, King Fahd University of Petroleum and Minerals, Dhahran 31261, Saudi Arabia; c Department of Chemistry, Islamia University, Bahawalpur, Pakistan; d Department of Chemistry, Government College College University, Lahore 54000, Pakistan; e Department of Chemistry, University of Engineering and Technology, Lahore 54890, Pakistan

## Abstract

The asymmetric unit of the title compound, (C_6_H_18_N_2_)[ZnCl_4_], consists of one tetra­chlorido­zincate anion and two half-*N*,*N*,*N*′*N*′-tetra­methyl­ethylenedi­ammonium cations. Each of the two di­ammonium cations is located about an inversion center and one of them is disordered over two sets of sites in a 0.780 (17):0.220 (17) ratio. The Zn^II^ atom has a slightly distorted tetra­hedral coordination environment. The cations and anions are connected *via* N—H⋯Cl hydrogen bonds into chains extending along [0-11].

## Related literature

For background to organic–inorganic hybrid materials, see: Al-Ktaifani & Rukiah (2011 ▶). For the isotypic tetra­chlorido­cobaltate(II) salt, see: Baughman *et al.* (2011 ▶). For other related structures and discussion of geometrical features, see: Yin & Wu (2010 ▶); Zhao & Qu (2010 ▶).
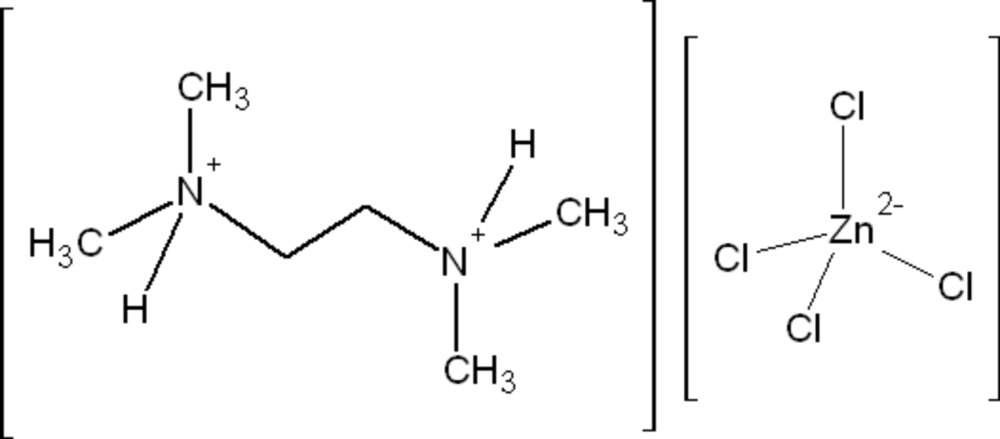


## Experimental

### 

#### Crystal data

(C_6_H_18_N_2_)[ZnCl_4_]
*M* *_r_* = 325.42
Triclinic, 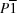

*a* = 6.893 (4) Å
*b* = 8.257 (6) Å
*c* = 13.33 (1) Å
α = 72.78 (3)°
β = 87.44 (3)°
γ = 69.42 (3)°
*V* = 676.9 (8) Å^3^
*Z* = 2
Mo *K*α radiation
μ = 2.57 mm^−1^
*T* = 293 K
0.95 × 0.44 × 0.08 mm

#### Data collection

Bruker SMART APEX area-detector diffractometer
Absorption correction: analytical (*SADABS*; Sheldrick, 1996 ▶) *T*_min_ = 0.194, *T*_max_ = 0.821
9440 measured reflections
2466 independent reflections
1806 reflections with *I* > 2σ(*I*)
*R*_int_ = 0.041

#### Refinement

*R*[*F* ^2^ > 2σ(*F* ^2^)] = 0.031
*wR*(*F* ^2^) = 0.075
*S* = 1.05
2466 reflections
154 parameters
6 restraints
H atoms treated by a mixture of independent and constrained refinement
Δρ_max_ = 0.40 e Å^−3^
Δρ_min_ = −0.54 e Å^−3^

### 

Data collection: *APEX2* (Bruker, 2007 ▶); cell refinement: *SAINT* (Bruker, 2007 ▶); data reduction: *SAINT*; program(s) used to solve structure: *SHELXS97* (Sheldrick, 2008 ▶); program(s) used to refine structure: *SHELXL97* (Sheldrick, 2008 ▶); molecular graphics: *ORTEP-3 for Windows* (Farrugia, 2012 ▶) and *Mercury* (Macrae *et al.*, 2008 ▶); software used to prepare material for publication: *SHELXL97*.

## Supplementary Material

Crystal structure: contains datablock(s) I, New_Global_Publ_Block. DOI: 10.1107/S1600536813029802/gk2591sup1.cif

Structure factors: contains datablock(s) I. DOI: 10.1107/S1600536813029802/gk2591Isup2.hkl

Additional supplementary materials:  crystallographic information; 3D view; checkCIF report

## Figures and Tables

**Table 1:** Hydrogen-bond geometry (Å, °)

*D*—H⋯*A*	*D*—H	H⋯*A*	*D*⋯*A*	*D*—H⋯*A*
N2—H2*N*⋯Cl1	0.91	2.30	3.157 (3)	158
N1—H1*N*⋯Cl3^i^	0.85 (4)	2.44 (4)	3.227 (4)	155 (3)

Symmetry code: (i) 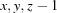.

**Fig. 1.**
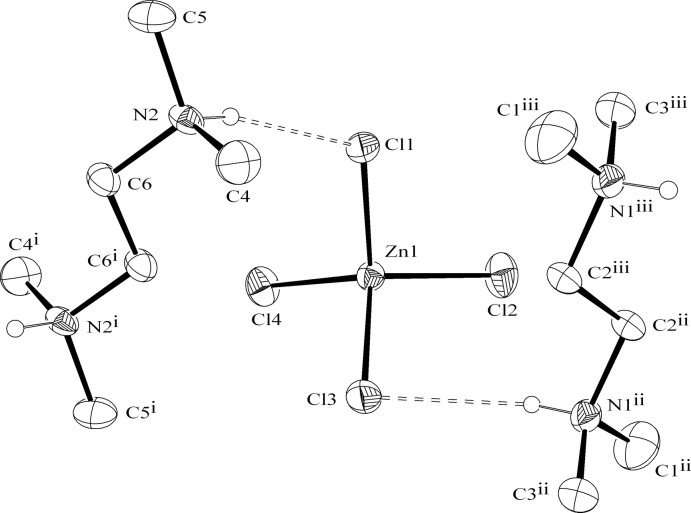
Molecular structure of the title compound showing the atomic numbering scheme [symmetry codes: (i) 1 - x,2 - y,1 - z; (ii) 1 - x,1 - y,1 - z; (iii) x,y,1 + z]. Displacement ellipsoids are drawn at the 30% probability level. For clarity, only N-H group H atoms are shown.

**Fig. 2.**
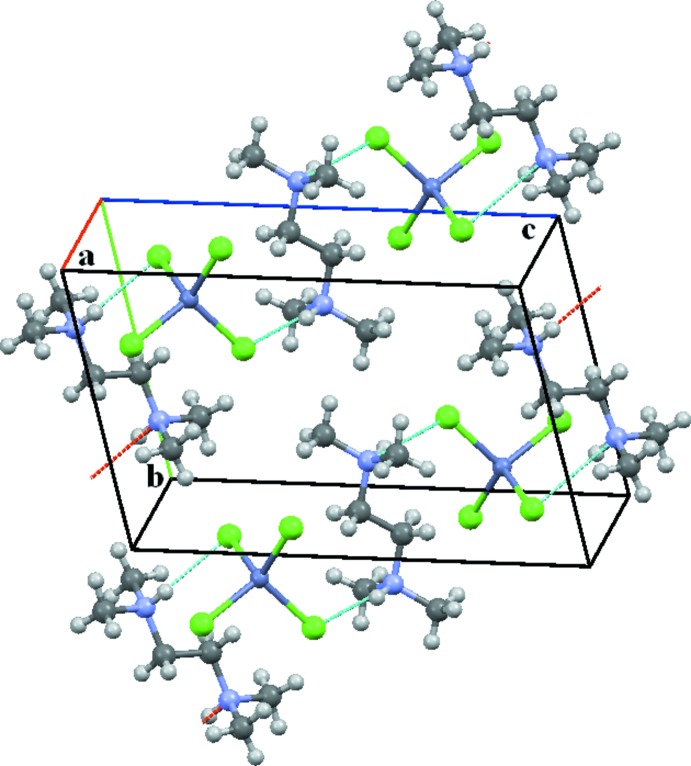
Packing diagram of the title compound showing the hydrogen-bonding interactions.

## supplementary crystallographic information

### 1. Comment

The organic-inorganic hybrid salts consisting of organic cation and polyhalometal counter anions have received considerable attention because of their potential applications in analytical, material and supramolecular chemistry (Al-Ktaifani *et al.*, 2011). In this work, we report the crystal structure of one such compound. The title compound was obtained unexpectedly during an attempt to synthesize a mixed-ligand zinc(II) complex of *N*,*N*,*N*'*N*'-tetramethylethylenediamine and 2-mercaptosuccinic acid. The crystal structure of the title hybrid material is shown in Fig. 1. The structure is ionic and the asymmetric unit consists of one tetrachloridozincate anion and two halves of tetramethylethylenediammonium cations, located on inversion centers. The Zn atom, coordinated by four chloride anions, shows a distorted tetrahedral environment (Fig. 1). The bond angles around zinc vary from 105.93 (7)° to 115.80 (7)°. The compound is isostructural with its cobalt analogue (Baughman *et al.*, 2011). In the crystal,the cations and anions are linked by N—H···Cl hydrogen bonds into chains propagating along the [011] direction (Fig. 2).

### 2. Experimental

The title complex was prepared by adding 0.12 g (1.0 mmol) of *N*,*N*,*N*'*N*'-tetramethylethylenediamine in 10 ml methanol to an aqueous solution (5 ml) of 0.14 g (1.0 mmol) zinc chloride. The slightly turbid solution was stirred for 15 minutes. Then a solution of 0.16 g (1.0 mmol) 2-mercaptosuccinic acid in 15 ml was added. The mixture was stirred for 30 minutes along with heating. The white mixture obtained was filtered and the filtrate was kept at room temperature for crystallization. As a result, white crystalline product was obtained, that was washed with methanol.

### 3. Refinement

The H1N atom of one of the symmetry independent cations was located on a difference Fourier map and freely refined. All other H atoms were placed in calculated positions with a C—H distance of 0.96 Å (*U*_iso_(H) = 1.5*U*_eq_(C)) for methyl groups, 0.97 Å (*U*_iso_(H) = 1.2*U*_eq_(C)) for methylene groups and N—H distance of 0.91 Å (*U*_iso_(H) = 1.2*U*_eq_(H)) for the other NH group. One of the diammonium cations the atoms C1, C2 and C3 are disordered over two positions, with the occupancy of the major position of 0.780 (17). During the refinement process restraints were imposed on C—N bond distances in the disordered cation.

### Crystal data

**Table:**

(C_6_H_18_N_2_)[ZnCl_4_]	*Z* = 2
*M**_r_* = 325.42	*F*(000) = 332
Triclinic, *P*1	*D*_x_ = 1.596 Mg m^−^^3^
Hall symbol: -P 1	Mo *K*α radiation, λ = 0.71073 Å
*a* = 6.893 (4) Å	Cell parameters from 162 reflections
*b* = 8.257 (6) Å	θ = 3.2–25.6°
*c* = 13.33 (1) Å	µ = 2.57 mm^−^^1^
α = 72.78 (3)°	*T* = 293 K
β = 87.44 (3)°	Needle, colorless
γ = 69.42 (3)°	0.95 × 0.44 × 0.08 mm
*V* = 676.9 (8) Å^3^	

### Data collection

**Table:**

Bruker SMART APEX area-detector diffractometer	2466 independent reflections
Radiation source: fine-focus sealed tube	1806 reflections with *I* > 2σ(*I*)
Graphite monochromator	*R*_int_ = 0.041
ω scans	θ_max_ = 25.6°, θ_min_ = 3.2°
Absorption correction: analytical (*SADABS*; Sheldrick, 1996)	*h* = −8→8
*T*_min_ = 0.194, *T*_max_ = 0.821	*k* = −9→9
9440 measured reflections	*l* = −15→16

### Refinement

**Table:**

Refinement on *F*^2^	Primary atom site location: structure-invariant direct methods
Least-squares matrix: full	Secondary atom site location: difference Fourier map
*R*[*F*^2^ > 2σ(*F*^2^)] = 0.031	Hydrogen site location: inferred from neighbouring sites
*wR*(*F*^2^) = 0.075	H atoms treated by a mixture of independent and constrained refinement
*S* = 1.05	*w* = 1/[σ^2^(*F*_o_^2^) + (0.0336*P*)^2^ + 0.066*P*] where *P* = (*F*_o_^2^ + 2*F*_c_^2^)/3
2466 reflections	(Δ/σ)_max_ = 0.001
154 parameters	Δρ_max_ = 0.40 e Å^−^^3^
6 restraints	Δρ_min_ = −0.54 e Å^−^^3^

### Special details

**Table:**

Geometry. All esds (except the esd in the dihedral angle between two l.s. planes) are estimated using the full covariance matrix. The cell esds are taken into account individually in the estimation of esds in distances, angles and torsion angles; correlations between esds in cell parameters are only used when they are defined by crystal symmetry. An approximate (isotropic) treatment of cell esds is used for estimating esds involving l.s. planes.
Refinement. Refinement of *F*^2^ against ALL reflections. The weighted *R*-factor *wR* and goodness of fit *S* are based on *F*^2^, conventional *R*-factors *R* are based on *F*, with *F* set to zero for negative *F*^2^. The threshold expression of *F*^2^ > σ(*F*^2^) is used only for calculating *R*-factors(gt) *etc*. and is not relevant to the choice of reflections for refinement. *R*-factors based on *F*^2^ are statistically about twice as large as those based on *F*, and *R*- factors based on ALL data will be even larger.

### Fractional atomic coordinates and isotropic or equivalent isotropic displacement parameters (Å^2^)

**Table:**

	*x*	*y*	*z*	*U*_iso_*/*U*_eq_	Occ. (<1)
Zn1	0.08487 (5)	0.89099 (5)	0.74635 (3)	0.03316 (14)	
Cl1	0.28956 (12)	0.66541 (11)	0.67979 (6)	0.0410 (2)	
Cl2	−0.07173 (14)	0.75755 (15)	0.87904 (8)	0.0654 (3)	
Cl3	0.30346 (14)	0.98281 (13)	0.81654 (7)	0.0518 (3)	
Cl4	−0.12777 (13)	1.13302 (12)	0.62136 (8)	0.0541 (3)	
N2	0.6233 (4)	0.7466 (3)	0.5224 (2)	0.0339 (6)	
H2N	0.5080	0.7274	0.5510	0.041*	
C1	0.178 (2)	0.727 (2)	0.1182 (13)	0.080 (4)	0.780 (17)
H1A	0.2288	0.6592	0.1898	0.120*	0.780 (17)
H1B	0.0775	0.6846	0.0974	0.120*	0.780 (17)
H1C	0.1146	0.8531	0.1123	0.120*	0.780 (17)
N1	0.3533 (4)	0.7001 (4)	0.0490 (2)	0.0433 (8)	0.50
H1N	0.301 (6)	0.773 (5)	−0.011 (3)	0.067 (13)*	
C2	0.4241 (11)	0.5105 (4)	0.0424 (4)	0.0374 (19)	0.780 (17)
H2A	0.3053	0.4832	0.0264	0.045*	0.780 (17)
H2B	0.4901	0.4258	0.1096	0.045*	0.780 (17)
C3	0.5165 (17)	0.756 (2)	0.0811 (17)	0.064 (3)	0.780 (17)
H3A	0.4547	0.8759	0.0881	0.096*	0.780 (17)
H3B	0.6167	0.7563	0.0287	0.096*	0.780 (17)
H3C	0.5841	0.6728	0.1473	0.096*	0.780 (17)
C1'	0.210 (7)	0.655 (7)	0.129 (4)	0.081 (15)	0.220 (17)
H1'1	0.0977	0.7635	0.1299	0.121*	0.220 (17)
H1'2	0.2835	0.5981	0.1972	0.121*	0.220 (17)
H1'3	0.1554	0.5729	0.1124	0.121*	0.220 (17)
N1'	0.3533 (4)	0.7001 (4)	0.0490 (2)	0.0433 (8)	0.50
C2'	0.534 (3)	0.545 (2)	0.0335 (14)	0.041 (7)	0.220 (17)
H2'1	0.5918	0.4579	0.1013	0.049*	0.220 (17)
H2'2	0.6407	0.5894	−0.0001	0.049*	0.220 (17)
C3'	0.454 (8)	0.799 (10)	0.092 (7)	0.087 (18)	0.220 (17)
H3'1	0.3489	0.9006	0.1069	0.130*	0.220 (17)
H3'2	0.5422	0.8416	0.0418	0.130*	0.220 (17)
H3'3	0.5349	0.7194	0.1560	0.130*	0.220 (17)
C4	0.7855 (5)	0.6795 (5)	0.6093 (3)	0.0564 (11)	
H4A	0.8210	0.5510	0.6401	0.085*	
H4B	0.7337	0.7393	0.6619	0.085*	
H4C	0.9066	0.7046	0.5823	0.085*	
C5	0.6891 (5)	0.6428 (5)	0.4463 (3)	0.0529 (10)	
H5A	0.8031	0.6690	0.4101	0.079*	
H5B	0.5752	0.6764	0.3963	0.079*	
H5C	0.7318	0.5155	0.4829	0.079*	
C6	0.5632 (5)	0.9456 (4)	0.4667 (3)	0.0393 (8)	
H6A	0.6874	0.9753	0.4499	0.047*	
H6B	0.4840	0.9756	0.4012	0.047*	

### Atomic displacement parameters (Å^2^)

**Table:**

	*U*^11^	*U*^22^	*U*^33^	*U*^12^	*U*^13^	*U*^23^
Zn1	0.0325 (2)	0.0332 (2)	0.0319 (2)	−0.00986 (16)	0.00269 (15)	−0.00935 (18)
Cl1	0.0448 (5)	0.0389 (5)	0.0395 (5)	−0.0121 (4)	0.0087 (4)	−0.0165 (4)
Cl2	0.0617 (6)	0.0736 (7)	0.0589 (7)	−0.0331 (5)	0.0258 (5)	−0.0092 (6)
Cl3	0.0660 (6)	0.0532 (6)	0.0450 (6)	−0.0335 (5)	−0.0084 (4)	−0.0108 (5)
Cl4	0.0452 (5)	0.0446 (6)	0.0555 (6)	−0.0068 (4)	−0.0114 (4)	0.0002 (5)
N2	0.0295 (13)	0.0262 (15)	0.0493 (18)	−0.0108 (11)	0.0103 (12)	−0.0161 (14)
C1	0.075 (5)	0.091 (8)	0.068 (6)	−0.017 (6)	0.025 (4)	−0.033 (6)
N1	0.0572 (19)	0.0316 (18)	0.0320 (18)	−0.0055 (14)	−0.0015 (15)	−0.0083 (16)
C2	0.041 (3)	0.030 (3)	0.045 (3)	−0.017 (2)	0.004 (3)	−0.012 (2)
C3	0.096 (8)	0.055 (5)	0.054 (6)	−0.042 (6)	−0.003 (6)	−0.016 (4)
C1'	0.06 (2)	0.13 (5)	0.08 (3)	−0.05 (2)	0.025 (19)	−0.06 (3)
N1'	0.0572 (19)	0.0316 (18)	0.0320 (18)	−0.0055 (14)	−0.0015 (15)	−0.0083 (16)
C2'	0.059 (13)	0.029 (10)	0.039 (12)	−0.015 (9)	−0.018 (10)	−0.014 (9)
C3'	0.08 (2)	0.12 (5)	0.08 (3)	−0.07 (3)	0.01 (3)	−0.02 (3)
C4	0.054 (2)	0.049 (2)	0.054 (3)	−0.0059 (18)	−0.0111 (19)	−0.011 (2)
C5	0.050 (2)	0.045 (2)	0.067 (3)	−0.0097 (18)	0.0089 (19)	−0.033 (2)
C6	0.0411 (18)	0.0296 (19)	0.046 (2)	−0.0115 (14)	0.0103 (15)	−0.0118 (17)

### Geometric parameters (Å, º)

**Table:**

Zn1—Cl2	2.2404 (15)	C3—H3C	0.9600
Zn1—Cl3	2.2516 (13)	C1'—H1'1	0.9600
Zn1—Cl4	2.2596 (17)	C1'—H1'2	0.9600
Zn1—Cl1	2.2949 (16)	C1'—H1'3	0.9600
N2—C5	1.472 (4)	C2'—C2'^i^	1.50 (5)
N2—C4	1.483 (4)	C2'—H2'1	0.9700
N2—C6	1.500 (4)	C2'—H2'2	0.9700
N2—H2N	0.9100	C3'—H3'1	0.9600
C1—N1	1.481 (2)	C3'—H3'2	0.9600
C1—H1A	0.9600	C3'—H3'3	0.9600
C1—H1B	0.9600	C4—H4A	0.9600
C1—H1C	0.9600	C4—H4B	0.9600
N1—C3	1.480 (2)	C4—H4C	0.9600
N1—C2	1.496 (2)	C5—H5A	0.9600
N1—H1N	0.85 (4)	C5—H5B	0.9600
C2—C2^i^	1.511 (13)	C5—H5C	0.9600
C2—H2A	0.9700	C6—C6^ii^	1.486 (6)
C2—H2B	0.9700	C6—H6A	0.9700
C3—H3A	0.9600	C6—H6B	0.9700
C3—H3B	0.9600		
			
Cl2—Zn1—Cl3	107.45 (6)	C2^i^—C2—H2A	109.7
Cl2—Zn1—Cl4	115.80 (6)	N1—C2—H2B	109.7
Cl3—Zn1—Cl4	108.07 (6)	C2^i^—C2—H2B	109.7
Cl2—Zn1—Cl1	105.93 (7)	H2A—C2—H2B	108.2
Cl3—Zn1—Cl1	106.21 (6)	H1'1—C1'—H1'2	109.5
Cl4—Zn1—Cl1	112.86 (7)	H1'1—C1'—H1'3	109.5
C5—N2—C4	110.7 (3)	H1'2—C1'—H1'3	109.5
C5—N2—C6	110.0 (3)	C2'^i^—C2'—H2'1	109.6
C4—N2—C6	113.4 (3)	C2'^i^—C2'—H2'2	109.6
C5—N2—H2N	107.5	H2'1—C2'—H2'2	108.1
C4—N2—H2N	107.5	H3'1—C3'—H3'2	109.5
C6—N2—H2N	107.5	H3'1—C3'—H3'3	109.5
C3—N1—C1	111.3 (11)	H3'2—C3'—H3'3	109.5
C3—N1—C2	115.7 (6)	C6^ii^—C6—N2	110.7 (3)
C1—N1—C2	108.9 (7)	C6^ii^—C6—H6A	109.5
C3—N1—H1N	107 (3)	N2—C6—H6A	109.5
C1—N1—H1N	105 (3)	C6^ii^—C6—H6B	109.5
C2—N1—H1N	108 (3)	N2—C6—H6B	109.5
N1—C2—C2^i^	110.0 (5)	H6A—C6—H6B	108.1
N1—C2—H2A	109.7		

Symmetry codes: (i) −*x*+1, −*y*+1, −*z*; (ii) −*x*+1, −*y*+2, −*z*+1.

### Hydrogen-bond geometry (Å, º)

**Table:**

*D*—H···*A*	*D*—H	H···*A*	*D*···*A*	*D*—H···*A*
N2—H2*N*···Cl1	0.91	2.30	3.157 (3)	158
N1—H1*N*···Cl3^iii^	0.85 (4)	2.44 (4)	3.227 (4)	155 (3)

Symmetry code: (iii) *x*, *y*, *z*−1.

## References

[bb1] Al-Ktaifani, M. M. & Rukiah, M. K. (2011). *Chem. Pap.* **65**, 469–476.

[bb2] Baughman, R. G., Shane, R. S. & McCormick, J. M. (2011). *Acta Cryst.* E**67**, m1. 10.1107/S1600536810048749 PMC3050123 21522515

[bb3] Bruker (2007). *APEX2* and *SAINT* Bruker AXS Inc., Madison, Wisconsin, USA.

[bb4] Farrugia, L. J. (2012). *J. Appl. Cryst.* **45**, 849–854.

[bb5] Macrae, C. F., Bruno, I. J., Chisholm, J. A., Edgington, P. R., McCabe, P., Pidcock, E., Rodriguez-Monge, L., Taylor, R., van de Streek, J. & Wood, P. A. (2008). *J. Appl. Cryst.* **41**, 466–470.

[bb7] Sheldrick, G. M. (1996). *SADABS* University of Göttingen, Germany.

[bb6] Sheldrick, G. M. (2008). *Acta Cryst.* A**64**, 112–122. 10.1107/S0108767307043930 18156677

[bb8] Yin, M. & Wu, S.-T. (2010). *Acta Cryst.* E**66**, m515. 10.1107/S1600536810012547 PMC2979142 21579012

[bb9] Zhao, M. M. & Qu, Z. R. (2010). *Acta Cryst.* C**66**, m188–m190. 10.1107/S0108270110022201 20603553

